# Social support as a mediator between sleep disturbances, depressive symptoms, and health-related quality of life in patients undergoing hemodialysis

**DOI:** 10.1371/journal.pone.0216045

**Published:** 2019-04-29

**Authors:** Kuei-Ching Pan, Shih-Yuan Hung, Chun-I Chen, Chu-Yun Lu, Mei-Ling Shih, Chiung-Yu Huang

**Affiliations:** 1 Department of Nursing, BenQ Medical Center, The Affiliated BenQ Hospital of Nanjing Medical University, Nanjing, Jiangsu Province, China; 2 Department of Nephrology, E-Da Hospital, Kaohsiung, Taiwan; 3 College of Medicine, I-Shou University, Kaohsiung, Taiwan; 4 Department of Industrial Management, I-Shou University, Kaohsiung, Taiwan; 5 Department of Nursing, I-Shou University, Kaohsiung, Taiwan; 6 Department of Nursing, E-Da Hospital, Kaohsiung, Taiwan; Fordham University, UNITED STATES

## Abstract

**Background:**

The hemodialysis regimen is an inevitable and mandatory treatment for patients with end-stage renal disease (ESRD). During the dialysis journey, patients may experience maladaptation in terms of sleep disturbances, depressive symptoms, and reduced health-related quality of life (HRQOL). Psychosocial resources such as social support may have beneficial influences on health outcomes, but studies have rarely analyzed the integrated relationships among risk factors which include pain, sleep disturbances, duration since diagnosis and various health outcomes in Taiwan. This study aimed to bridge this gap by investigating the relationships among related risk factors, social support, sleep disturbances, depressive symptoms, and HRQOL, which is composed of physical quality of life (PQOL) and mental quality of life (MQOL), in ESRD patients.

**Method:**

A correlational design was used, and 178 patients aged 20 years or older were recruited via convenience sample. The relationships among the risk factors, the mediators, depressive symptoms, PQOL, and MQOL were analyzed using structural equation modeling.

**Results:**

The findings showed that more than 70% of the participants reported poor sleep quality, and 32% reported depressive symptoms. When participants had greater pain and more sleep disorders, they were more likely to be depressed. When participants had more appraisal support; they had better PQOL and fewer depressive symptoms. Overall, the structural equation model explained 31.8% of the variance in self-reported depressive symptoms, 29.4% of the variance in PQOL, and 5.7% of the variance in MQOL. Moreover, appraisal support enhanced PQOL and reduced depressive symptoms by exerting its two mediating effects on sleep disturbances.

**Conclusion:**

Our findings indicate that patients with ESRD who have more social support have better PQOL and MQOL and fewer depressive symptoms than those with less social support.

## Introduction

End-stage renal disease (ESRD), which is highly prevalent worldwide, is a complication of the primary disease of diabetes or the cardiovascular system [1], and patients must accept permanent dialysis for the remainder of their lives if they do not accept further aggressive treatment such as kidney transplant. Psychological problems may occur when patients with ESRD undergo long-term dialysis, and depressive symptoms have been reported to be highly prevalent in patients with ESRD [2, 3]. Moreover, the loss of bodily control among patients with ESRD is accompanied by depressive symptoms and results in negative outcomes in terms of economic burden, family dysfunction, and worse health-related quality of life (HRQOL) [4–8].

In the US, approximately 20–30% of patients with ESRD have significant depressive symptoms, which could contribute to distress and sleep disturbances [9–12] and increase the risk of mortality and morbidity. In Taiwan, the prevalence of depressive symptoms in patients with ESRD is relatively high at approximately 50–70% [13–15]. Moreover, sleep disturbances are frequently reported in patients with ESRD, which may be due to symptoms related to uremia-associated sleep disturbances or renal-related symptoms or treatments [11, 16].

The literature has indicated that having sufficient social support resources may reduce emotional stress and help enhance adaptation skills in daily life among patients with spinal cord injury [17], mental health issues [18], osteoporosis [19], and breast cancer [20], but few studies have mentioned the effect of social support on patients with ESRD in Taiwan. Social support is multidimensional, and four aspects have often been evaluated in different fields: informational support (IS), emotional support (ES), appraisal support (AS), and tangible support (TS) [21]. This study used the modified Chinese version of the Social Support Inventory (SSI) [22], which has been found to have good reliability, to assess social support. The SSI dimensions include ES, which focuses on individual behaviors intended to support people, including love and empathy. IS refers to suggestions and information provided to help people make treatment decisions or deal with life events. TS consists of material assistance, and AS refers to affirmations of (or respect for) an individuals’ activities, values, or progression. Each of these categories of social support plays an important role in meeting the needs of patients with ESRD and their families. Previous studies [23, 24] in different fields have suggested that individuals who perceive high levels of social support experience better quality of life (QOL), leading to enhanced well-being [8, 19].

Numerous studies have suggested that social support may have mediating or moderating effects on health. Further, research has shown that a lack of social support is related to depressive symptoms, anxiety, frustration, and social withdrawal [17, 25]. Too little research has focused on determining the effects of different types of social support on depression in patients with ESRD in Taiwan, especially the integrated relationships among disease factors and the impact of social support on depression in patients with ESRD. Baron and Kenny [26] defined mediation as “the generative mechanism through which the focal independent variable is able to influence the dependent variable of interest.” Mediation is used to assess hypotheses in which the main independent variable operates through a mediator to impact a dependent variable [26]. Therefore, structural equation modeling (SEM) was applied to examine the relationships between the risk factors which include pain, sleep disturbances, duration since diagnosis and the outcomes. HRQOL was measured based on physical quality of life (PQOL) and mental quality of life (MQOL), which represent well-being. To clarify the types of social support and to show that HRQOL is associated with risk factors in patients with ESRD, the following research questions were proposed:

1. What are the relationships among risk factors which include pain, sleep disturbances, duration since diagnosis, types of social support, and depressive symptoms/PQOL/MQOL in patients with ESRD?
2. Does type of social support play a mediating role between sleep disturbances and depressive symptoms/PQOL/MQOL in patients with ESRD?

## Materials and methods

### Research design

A cross-sectional survey was conducted, and a convenience sample of 178 patients with ESRD and eligible based on the below inclusion criteria was recruited from the hemodialysis center at a hospital, from August 2015 to Jan. 2016. Approval from the Institutional Review Board of E-Da Hospital (EMRP-103-098) was obtained for the study. Participants were provided information about the study. The inclusion criteria were as follows: 1.) aged 20 years or above, 2). undergoing routine hemodialysis treatment, and 3). able to communicate in Mandarin or Taiwanese. The exclusion criteria were as follows: 1). no DSM-IV psychiatric diagnoses, 2). no severe complications during dialysis, and 3). no other severe diseases such as cancer. After written informed consent was collected, the investigator conducted face-to-face interviews with a structured questionnaire. The recommended appropriate sample size for path analysis using SEM approaches is between 150 and 200 subjects [27]; thus, this study had an adequate sample size. A total of 185 eligible patients with ESRD were contacted, and seven eligible participants declined to participate because of time or fatigue. The response rate was 96%, and there were no missing data during the analysis.

### Variables and measures

The study used several questionnaires to measure the research variables, including demographics, pain, sleep quality, social support, depression, and HRQOL. The corresponding questionnaires included a demographic and clinical characteristics information sheet, the Visual Analogue Scale (VAS) [28], the Pittsburgh Sleep Quality Index (PSQI) [29], the SSI [22], the Center for Epidemiological Studies-Depression (CES-D) scale [30] and the Short Form-36 (SF-36) Health Survey [31].

A detailed description of each questionnaire is provided below.

#### Demographic and clinical characteristics

The demographic and clinical characteristics information sheet was self-reported and measured specific demographic variables, including age, duration since diagnosis, number of diseases which are the other diseases endorsed by participants chronic but well-controlled diseases, marital status, education, and monthly household income.

#### Visual analogue scale

Pain was measured by VAS, which is originally developed by Freyd in 1923 [28]. The VAS is widely used in pain assessment and has demonstrated good validity and reliability [32]. The VAS consists of a 10 cm long straight line with the two-side endpoints identifying as “no pain at all = 0” and “pain as bad as it could be = 10.” Individuals reported their pain level by marking on the line and the distance from zero to the marked point represents the pain score. A higher score indicates greater pain.

#### Pittsburgh sleep quality index

The PSQI was adopted to measure sleep quality [29]. The PSQI consists of 19 items that are divided into seven sections to evaluate participant perspectives of sleep quality. These sections are subjective sleep quality, sleep latency, sleep duration, habitual sleep efficiency, sleep disturbances, use of sleep medication, and daytime dysfunction over the last month. The global PSQI score of sleep quality was ranging from 0 to 21 with higher scores showing worse sleep quality. A PSQI score greater than 5 were deemed as sleep disturbances [29]. Cronbach’s α for the Kao et al.’s study was .82 [33] and was 0.78 in this study.

#### Social support inventory

Social support was assessed by a modified Chinese version of SSI [22], originally developed by Barrera, Sandler, and Ramsay [34], which has been used in evaluating patients with spinal cord injuries. The Cronbach’sαwas 0.86 in the previous study [17]. This 19-item scale applies a four-point Likert scale (1 = never to 4 = always) with total scores ranging from 19–76, includes emotional support (ES, Q1-4), informational support (IS, Q8-13), tangible support (TS, Q14-19), and Appraisal support (AS, Q5-7). The higher score indicates greater social support. The Cronbach’s α was 0.97 for the total support, in each subscale was 0.95 for ES, 0.95 for IS, 0.91 for AP, and 0.87 for TS in the current study.

#### Center for epidemiological studies depression

Depressive symptoms were evaluated by the CES-D scale [30]. The CES-D consists of 20-item scale, a 4-point scale ranging from 0 (= rarely or none of the time) to 3 (= most or almost all the time) in each item, and the total CES-D scores range from 0–60. The higher the scores the patients achieve, the more depressive symptoms the patients have. In this research, the Cronbach’s alpha was 0.84. The depressive level is classified as follows: scores lower than 16 show no depression; scores ranging from 16–20 show mild depressive symptoms; scores ranging from 21–26 show moderate depressive symptoms; and scores ranging from 27–60 show severe depressive symptoms [35].

#### SF-36 health survey

The HRQOL was measured by applying the SF-36 questionnaire, a generic indicator of health [31]. The SF-36 includes eight subscales relevant to the general health of the individual: physical function (PF), role physical (problems with work or other daily activities as a result of physical health; RP), bodily pain (BP), general health (GH), social functioning (SF), role-emotional (problems with work or other daily activities as a result of emotional problems; RE), vitality (VT), and mental health (MH). According to a previous study that suggested manual scoring [31], SF-36 is divided into two components: PQOL consisting of PF, RP, BP, GH; MQOL consisting of RE, VT, SF, and MH, and was employed in this manner for the study. Cronbach's α in the current study ranged from 0.71–0.85 among the subscales in this analysis.

### Data analysis

SEM was adopted to determine the effects of the risk factors which include pain, sleep disturbances, duration since diagnosis and social support on depressive symptoms/MQOL/PQOL using IBM SPSS AMOS 22.0. The goodness-of-fit index (GFI), average GFI (AGFI), and root mean square error of approximation (RMSEA) were calculated to assess the goodness-of-fit of the model. A model was considered to be a good fit if *X*^*2*^*/df* < 3 [27]. The values for GFI and AGFI should be ≧ 0.90, and the value for RMSEA should be ≦ 0.08. Additionally, the mediating effects of the four types of social support were examined using the methods described by Baron and Kenny [26] and Gogineni, Alsup, and Gillespie [36]. These authors suggested that complete mediation occurs when the effect of sleep disturbances on the outcome variables (depressive symptoms/PQOL/MQOL) is alleviated by the influence of social support as a mediating variable and is statistically not significant. If the result remains significant but the correlation decreases, then a partial mediating effect has occurred.

## Results

### Characteristics of the sample

A sample of 178 adults undergoing hemodialysis for ESRD participated in this study, and the mean age was 62.9 (*SD* = 11.5) years (Table 1). The average duration of hemodialysis was 56.8 (*SD* = 40.2) months, the mean pain level was 2.5 (*SD* = 1.3, score range 0–10), 159 participants had at least one chronic disease other than ESRD, most (58%) of the participants were males, and 136 (76.4%) of the participants were married/cohabitating. Most of the participants (70.8%) reported that their monthly household income was 25,000~75,000 per month in New Taiwan (NT) dollars (1 US dollar = 30.5 NT). The participants reported the following levels of education: illiterate (29, 16.3%), elementary (72, 40.4%), junior high (32, 18.1%), and college or above (12, 6.7%).

**Table 1:** Variables of sample (*n* = 178).

*Variables*	*Mean* (*SD*)	*n* (*%*)
**Age (years)**	62.9 (11.5)	
**Duration (months)**	56.8 (40.2)	
**Pain (1~10)**	2.5 (1.3)	
**Disease (numbers)**		
No other disease	19 (10.7)	
≧1	159 (89.3)	
**Marital status**		
Single		10 (5.6)
Married/cohabitating		136 (76.4)
Widowed		29 (16.3)
Divorced (other)		3 (1.7)
**School years (Education)**		
Illiterate		29 (16.3)
Elementary		72 (40.4)
Junior high		32 (18.1)
High school		33(18.5)
College (or above)		12 (6.7)
**Household income** **(Monthly NT dollars)**		
< 25,000		31 (17.4)
>25,000–50,000		66 (37.1)
>50,000–75,000		60 (33.7)
> 75,00–100,000		15 (8.4)
>100,000		6 (3.4)
**Sleep quality**	7.4 (4.1)	
**Depressive symptoms**	16.9 (5.5)	

Household income (monthly): New Taiwan (NT) Currency

### The prevalence of depressive symptoms and sleep quality

Table 1 shows that the average depressive symptom score (from the CES-D) was 16.9 (*SD* = 5.5, score range 1–43). Approximately 32% of the participants achieved the criteria for depressive symptoms on the CES-D scale; 29 participants (16.3%) were categorized as having mild depressive symptoms (score of 16–20), 15 (8.4%) had moderate depressive symptoms (score of 21–26), and 13 (7.3%) had severe depressive symptoms (score of 27–60). Approximately 107 participants (60%) reached the standard for sleep disturbances. The mean score for the sleep quality scale was 7.4 (*SD* = 4.1, range 0–17). Regarding the participants’ perspectives on sleep quality (Table 2), 117 participants (65.7%) were unsatisfied with their sleep quality. For approximately 71% of the participants, sleep latency was longer than 30 minutes, and sleep duration was less than or equal to 7 hours for 68% of the participants. One hundred twenty-five (70.2%) of the participants reported a habitual sleep efficiency of 75~84%, and most (75.3%) of the participants reported subjective sleep disturbances. Thirty-six percent of the patients used sleep aid medication, and 31.5% reported daytime dysfunction.

**Table 2:** Participants’ perspectives of sleep quality (*n* = 178).

*Items*	*n*	*%*
**Subjective sleep quality**		
Satisfied	61	34.3
Unsatisfied	117	65.7
**Sleep latency**		
0–30 minutes	51	28.6
31~60 minutes	43	24.2
>60 minute	81	47.2
**Sleep duration**		
>7Hrs	57	32
≦7Hrs	122	68
**Habitual sleep efficiency**		
75~84%	125	70.2
65~74%	53	29.8
**Subjective sleep disturbances**		
No	44	24.7
Yes	134	75.3
**Use of sleep medication**		
Use	64	36
Not use	114	64
**Daytime functioning**		
Yes	122	68.5
No	56	31.5
**Total scores of Sleep disturbances**		
>5	107	60.1
≦5	71	39.9

### Relationships among the variables

Table 3 shows that pain and sleep quality were positively correlated with depressive symptoms (*r* = 0.30, *p* < 0.01; *r* = 0.43, *p* < 0.01). The results indicated that those with higher levels of pain and sleep disturbance were more likely to be depressed. The less emotional support (*r* = -0.27, *p* < 0.01), appraisal support (*r* = -0.37, *p* < 0.01), informational support (*r* = -0.25, *p* < 0.01), and tangible support (*r* = -0.20, *p* < 0.01) the individuals perceived, the more depressive symptoms they reported. Depressive symptoms were negatively correlated with PQOL and MQOL (*r* = -.63, *p* < .01; *r* = -.34, *p* < .01). Moreover, when patients with ESRD had more pain, they reported worse PQOL (*r* = -.40, *p* < .01) and MQOL (*r* = -.21, *p* < .01). Patients with ESRD who reported sleep disturbances were more likely to have lower PQOL (*r* = -.26, *p* < .01), although there was no significant correlation with MQOL.

**Table 3:** Correlations among characteristics, social support, depressive symptoms, PQOL, MQOL (*n* = 178).

*Variables*	1	2	3	4	5	6	7	8	9	10	11	12
**1. Age**	1											
**2. Education**	-.43**											
**3. Income**	.03	.27**										
**4. Pain**	-.10	.03	-.12									
**5. Duration**	-.10	.042	.19*	-.10								
**6. Sleep quality**	.06	-.12	.00	.20**	-.02							
**7. ES**	.10	.03	.19*	.02	-.08	-.14						
**8. AS**	.07	.02	.22**	-.08	-.10	-.18*	.84**					
**9. IS**	.16*	-.09	.21**	-.02	-.08	-.10	.91**	.84**				
**10. TS**	.37**	-.19*	.25**	-.03	-.11	-.09	.78**	.67**	.80**			
**11. DSs**	.04	-.04	-.14	.30**	.00	.43**	-.27**	-.37**	-.25**	-.20**		
**12. PQOL**	-.19*	.24**	.12	-.40**	.05	-.26**	.07	.19**	.06	-.09	-.63**	
**13. MQOL**	.06	-.08	-.03	-.21**	.08	-.08	.06	.02	.06	-.01	-.34**	.66**

Note 1.

*: *p* < .05

**: *p* < .01

Note 2. ES = Emotional support; IS = Informational support; AS = Appraisal support; TS = Tangible support

DSs = Depressive symptoms; PQOL: Physical Quality of life; MQOL: Mental Quality of life.

### The constructs of risk factors, social support, and HRQOL/Depressive symptoms

**Fig 1:**
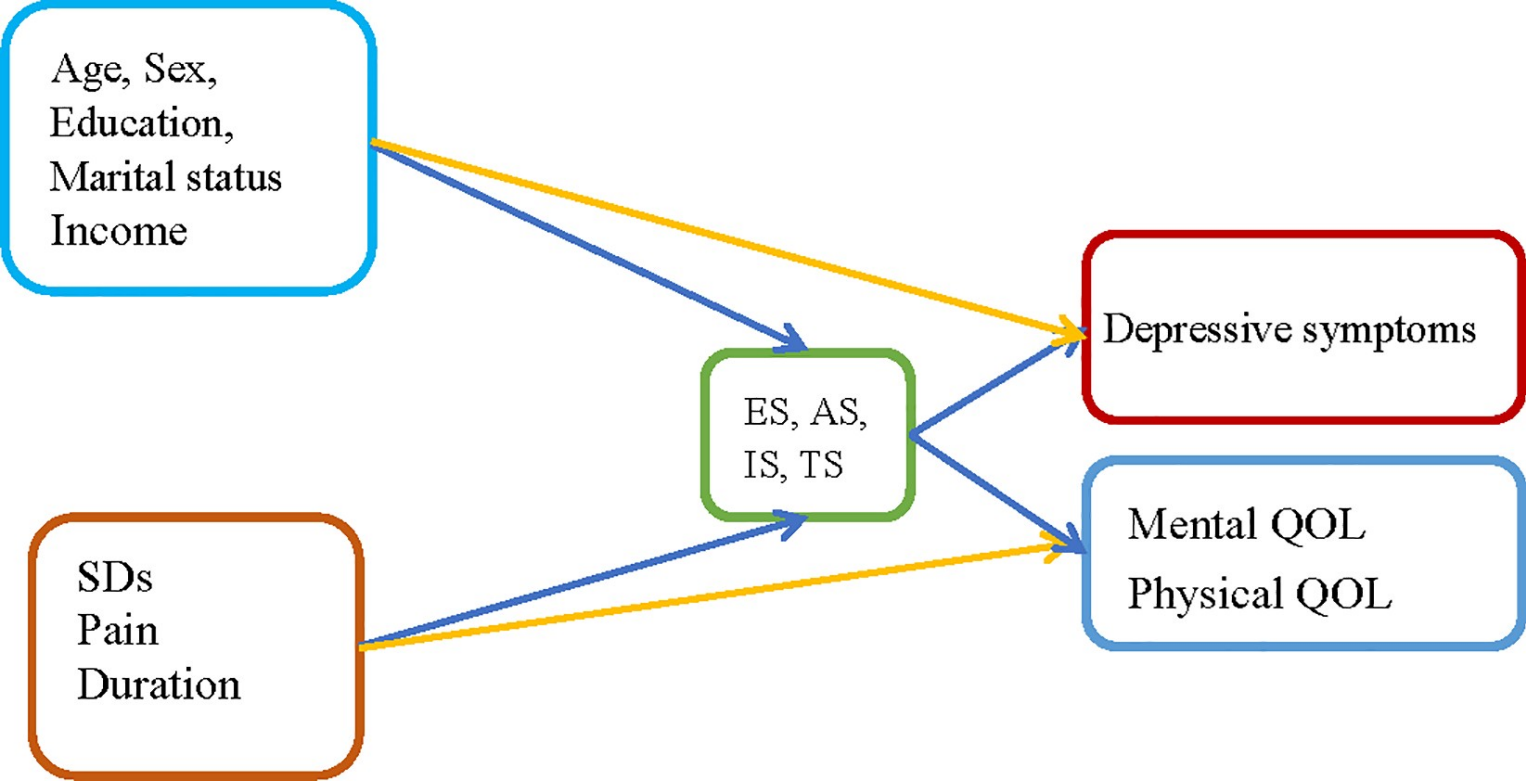
The path analysis of factors for patients with end-stage renal disease ESRD. Note: SDs: Sleep disturbances; ES: Emotional support; AS: Appraisal support; IS: Informational support; TS: Tangible support; QOL: Quality of life.

**Fig 2:**
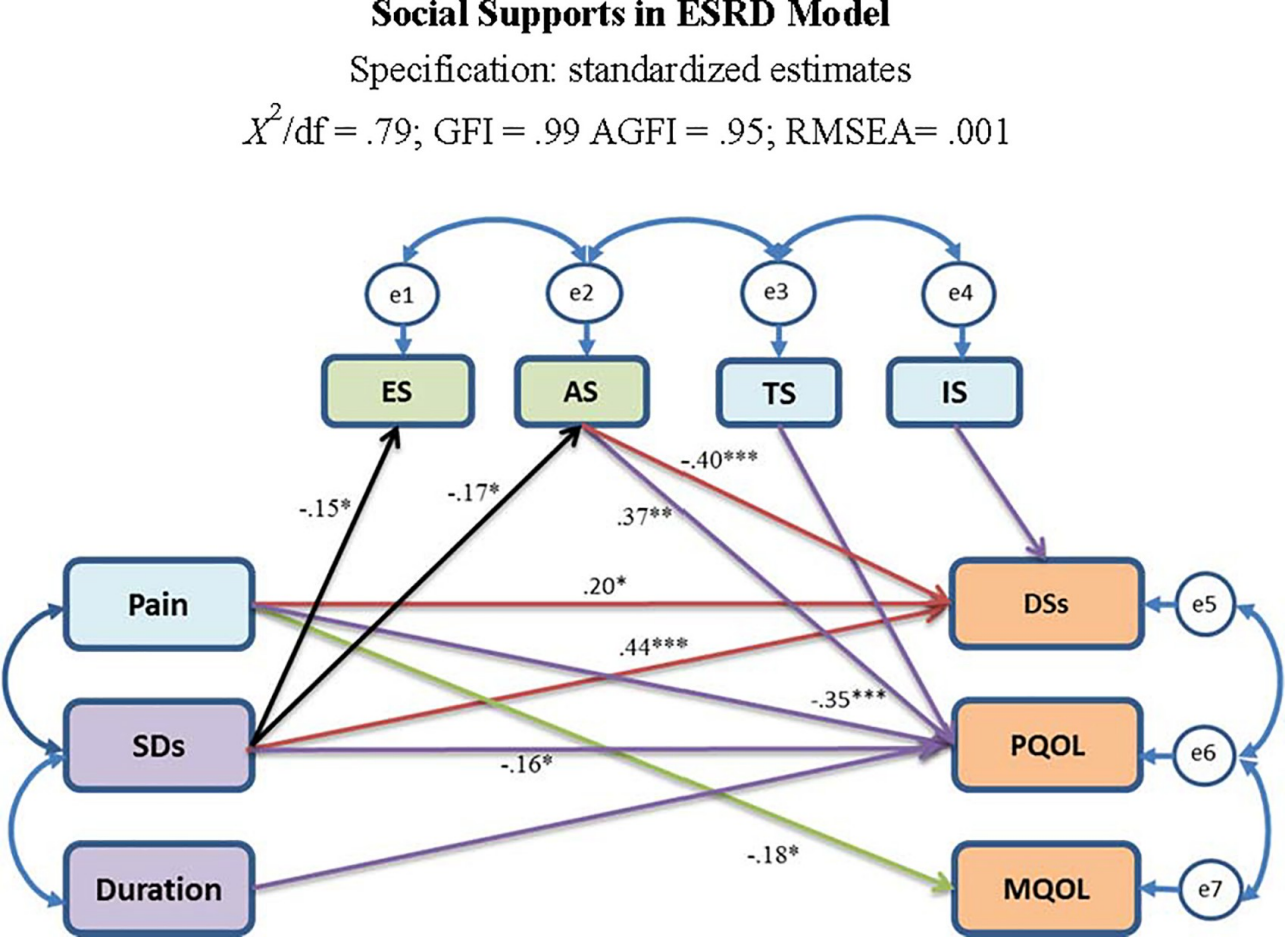
The SEM of predictors on depressive symptoms/PQOL/MQOL in patients with ESRD. **Notes** 1. SDs: Sleep disturbances; DSs: Depressive symptoms; ES: Emotional support; AS: Appraisal support; IS: Informational support; TS: Tangible support 2. * *p* < .05, **p < .01, ***p < .001.

**Fig 3:**
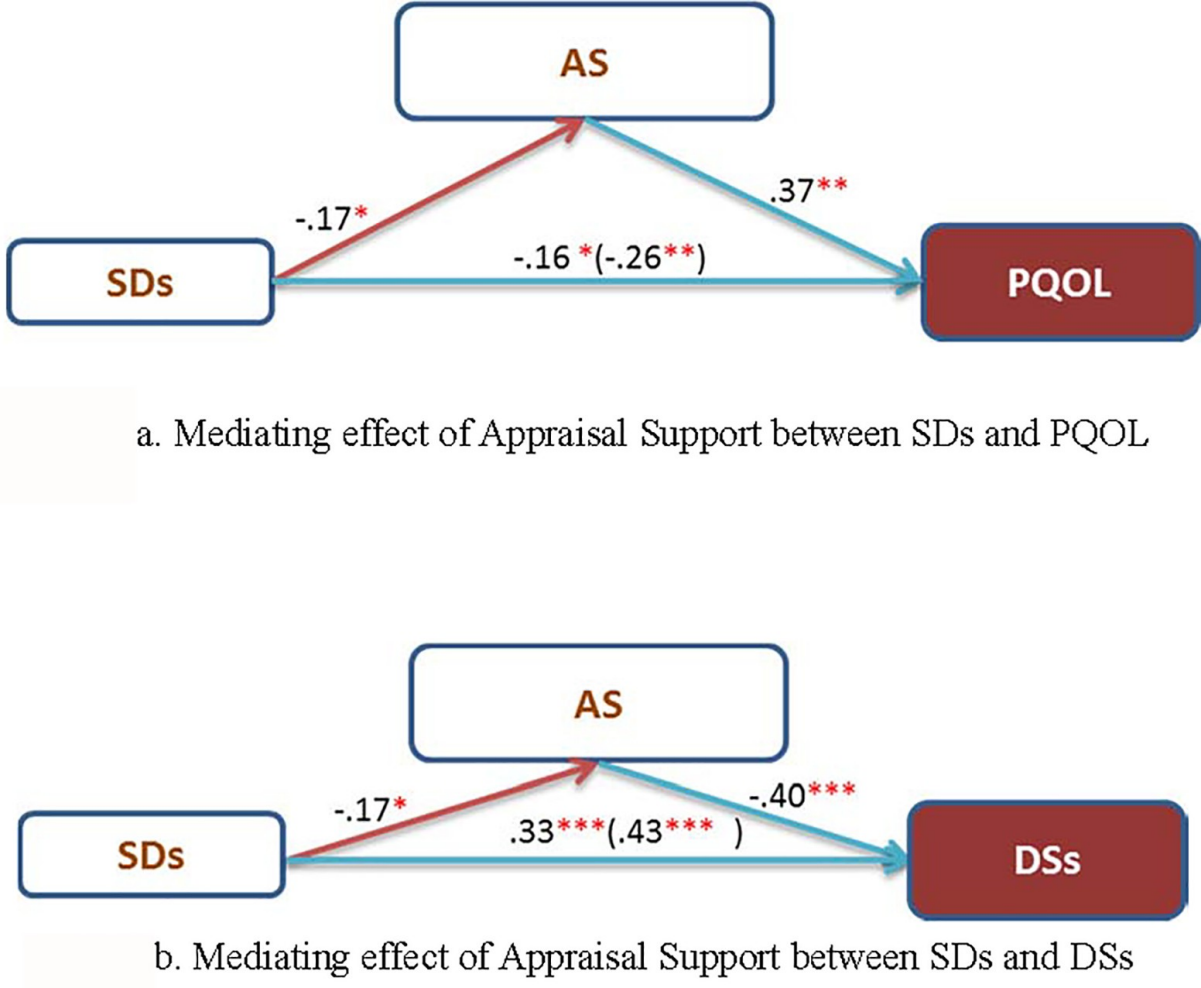
Mediating effects of appraisal support occurred on PQOL or DSs. Note. 1. SDs: Sleep disturbances, DSs: Depressive symptoms, AS: Appraisal support 2. *:*p* < .05, **:*p* < .01, ***:*p* < .001.

To examine the direct and indirect effects of the risk factors and social support on HRQOL and depressive symptoms, a model was constructed to evaluate the structure shown in Fig 1. Our findings showed that our initial conceptual model exhibited a lack of model fit. We examined a structural equation model to determine the direct/indirect effects of the four types of social support (ES, AS, IS, or TS) on health outcomes (PQOL, MQOL, and depressive symptoms) among patients with ESRD. After several modifications, the overall goodness-of-fit statistics revealed that the proposed model fit the data well, with RMSEA = 0.001, *χ*^2^/df = 0.79, GFI = 0.99, and AGFI = 0.95. Additionally, we observed partial mediating effects for the four types of social support. Fig 2 shows that this structural model consisted of independent variables (three subject characteristics, four types of social support) and three dependent variables: depressive symptoms, PQOL, and MQOL. Furthermore, the four types of social support were mediators of depressive symptoms/PQOL/MQOL. Appraisal support had a significantly negative direct effect on depressive symptoms (*β* = -0.40, *p* < 0.001), and higher levels of appraisal support led to better PQOL (*β* = 0.37, *p* < 0.01). The following mediating effects were observed: 1) Appraisal support mediated sleep disturbances and depressive symptoms, and 2) AS mediated sleep disturbances and PQOL (Fig 3). Overall, the structural model explained 31.8% of the variance in self-reported depressive symptoms, 29.4% of the variance in PQOL, and 5.7% of the variance in MQOL.

### Mediating effects of different types of social support

Specifically, among the four types of social support, only appraisal support played a mediating role between the risk factors and health outcomes: 1). Appraisal support mediated sleep disturbances and PQOL, and 2). Appraisal support mediated sleep disturbances and depressive symptoms (Fig 3). Fig 3A shows that appraisal support influenced sleep disturbances and PQOL. In step 1, sleep disturbances significantly affected PQOL (*β* = -.26, *p* < .001). In step 2, sleep disturbances significantly affected appraisal support (*β* = -.17, *p* < .05), and appraisal support affected PQOL (*β* = .37, *p* < .001). However, in step 3, the effect of sleep disturbances on PQOL was reduced but continued to be significant when appraisal support entered the regression (*β* = -.16, *p* < .05). Because only a reduction effect occurred, this result suggests that appraisal support only partially mediated the relationship between sleep disturbances and PQOL. Therefore, appraisal support can influence the relationship between sleep disturbances and PQOL. More specifically, the mediating effects of appraisal support can promote patient PQOL.

Fig 3B shows that appraisal support mediated the relationship between sleep disturbances and depressive symptoms. In step 1, sleep disturbances significantly influenced depressive symptoms (*β* = .43, *p* < .001). In step 2, sleep disturbances significantly influenced appraisal support (*β* = -.17, *p* < .05), and appraisal support affected depressive symptoms (*β* = -.40, *p* < .001). In step 3, when appraisal support entered the regression, the influence of sleep disturbances on depressive symptoms decreased (*β* = .33, *p* < .001) but remained significant. Therefore, appraisal support can change the relationship between sleep disturbances and depressive symptoms. Specifically, the mediating effects of appraisal support can lessen patients’ depressive symptoms.

## Discussion

This research focused on the relationships among individual demographic and clinical characteristics, including four types of social support, depressive symptoms, and HRQOL. The results showed that 32% of the participants reached the cut point for depressive symptoms. This result coincides with the findings of a previous study [17] that showed that the prevalence of depressive symptoms in patients with ESRD in long-term dialysis was 18–35% and that patients with more social support were less likely to experience depressive symptoms than those with less social support.

In this study, approximately 60% of patients with ESRD reported sleep disturbances, and sleep disturbances were positively correlated with depressive symptoms and negatively correlated with PQOL; these results are consistent with a previous study [14]. Sleep quality has been reported to be a health issue for 20–83% of patients with ESRD [11], and sleep quality is related to multiple factors. A further intervention study is needed to explore methods for decreasing sleep disturbances in patients with ESRD. One potential method is the use of psycho-education on how to enhance social support, which could lead to decreases in sleep disturbances. And this will need a further investigation to validate the causal relationship between social support and sleep quality in our future research. Based on the research results, the rates of depression and sleep disturbance coincided with previous studies on patients with ESRD. Due to geographic limitations that might affect the external validity of the research results, caution should be used when generalizing our results to a larger population.

Patients with ESRD showed a high percentage of depressive symptoms, possibly because patients have inadequate social support to meet their needs or tangible help for daily events during long-term dialysis. Additionally, participants may have pain or care information needs that are not being satisfied, which could result in worse HRQOL. In these circumstances, if ESRD patients have more access to social support, especially appraisal support, they may experience fewer depressive symptoms.

The literature has noted that satisfaction with social support can influence psychological outcomes [25]. As predicted in the correlational matrix, all four types of social support had a significant inverse relationship with depressive symptoms; however, after several modifications to achieve a good model fit, only appraisal support significantly influenced health outcomes. The reason why only appraisal support is remained after SEM analysis is possibly due to the confounding effect among four components of social support since they are mutually correlated. Thaden and Kneib [37] proposed that SEM techniques prove to be helpful to disentangle direct covariate effects from indirect covariate effects arising from correlation with other variables. There could be several reasons for the mediating effects of appraisal support in this study. First, appraisal support may act as a resource for patients as they cope with challenges. Second, there may be a mechanism through which greater appraisal support could enhance PQOL, i.e., by affirming individual activities or values. Finally, appraisal support may have an effect on individual adaptation to diagnosis, long-term dialysis, sleep disturbances, and other complications. The above explanations support the study findings regarding the mediating effects of appraisal support. Moreover, appraisal support may enhance patients’ self-esteem and reduce their feelings of frustration, and it may aid them in rebuilding their sense of well-being. This study also demonstrated an inverse relationship between appraisal support and depressive symptoms; this result explains why appraisal support provides protection against depression in the mediation model [38]. Through greater support, individuals can establish positive relationships that in turn mediate the effects of risk factors on health outcomes [39]; when only partial appraisal support is perceived, the degree of impact on negative health may be reduced.

Regarding the SEM results, only appraisal support played mediating roles between sleep disturbances and depressive symptoms/PQOL, while the other three types of social support had a correlational effect. Appraisal support may alleviate the detrimental effect of sleep disturbances through its mediating influence. Further investigation is needed regarding the reasons that the other types of social support did not play a mediating role in health outcomes.

The findings showed that appraisal support was significantly directly and indirectly associated with depressive symptoms in the current study, which is consistent with the results of previous studies [17]. Healthcare experts should implement psychosocial education to help patients with ESRD and their families obtain better access to resources and health-related information. Healthcare experts could organize support groups to improve positive self-efficacy, and they may need to provide social access and psychological support to enhance the level of mental support patients receive. Given differences in the physical conditions of patients, healthcare experts should concentrate on developing interventions related to self-efficacy that provide AS for patients in order to mitigate patients’ experience of severe depressive symptoms.

## Limitations

The study had some sampling and methodological limitations. First, convenience sampling was adopted, which might constrain the applicability of the research findings to the population in southern Taiwan; thus, generalizability may be limited. In the future, researchers can extend the research setting to different locations. Second, the subjective nature of self-reported questionnaires including social support or quality of life is also the concern. Third, the cross-sectional nature of the current study was a limitation, and the time effects of the study variables were unclear. Another limitation exists, regarding the exclusionary criteria; we exclude patients with pre-existing depression and serious medical disease, which may also limit the participants to attend this study. Therefore, it was not appropriate to develop inferences regarding the longitudinal influences of the independent variables on the HRQOL of patients with ESRD.

## Conclusion

The results revealed a high prevalence of depression and sleep disturbances in patients with ESRD in Taiwan, which is consistent with reported rates in Taiwan but higher than rates reported in the US. Social support played an important role as a mediator between sleep disturbances and depressive symptoms in patients with ESRD in this study. Our findings offer healthcare professionals a better understanding of ways to utilize social support, especially appraisal support, based on the finding that appraisal support promotes PQOL. Additionally, the findings provide general support for the hypotheses regarding the effect of social support on depressive symptoms. Further research should be carried out with nurses in renal departments to investigate their perceptions and knowledge of how to evaluate social support in patients with ESRD, which could lead to better health outcomes for patients with ESRD.
